# Role of social organization engagement in high-risk groups intervention against HIV/AIDS: a case study from 176 cities of China

**DOI:** 10.1186/s40249-022-01048-x

**Published:** 2022-12-28

**Authors:** Zhiwei Leng, Sha Sha, Shuyu Dai, Xuehui Meng, Jinfeng Li, Junyi Jin, Wenlin Zheng, Weihua Yang, Chuanju Mao, Zhenzhong Wang, Liujin Zhang, Peng Wang, Tao Yang, Weizhong Yang

**Affiliations:** 1 School of Population Medicine and Public Health, Chinese Academy of Medical Sciences & Peking Union Medical College, Beijing, China; 2 Chinese Preventive Medicine Association, Beijing, China; 3 Zhejiang Chinese Medical University, Hangzhou, China; 4 Centre for Health Management and Policy Research, School of Public Health, Cheeloo College of Medicine, Shandong University, Jinan, China; 5 Chinese Academy of Medical Sciences & Peking Union Medical College, Beijing, China

**Keywords:** Social organization, High-risk groups, HIV/AIDS, Spatial effect

## Abstract

**Background:**

A high-risk prevention strategy is an effective way to fight against human immunodeficiency virus (HIV) and acquired immunodeficiency syndrome (AIDS). The China AIDS Fund for Non-Governmental Organizations (CAFNGO) was established in 2015 to help social organizations intervene to protect high-risk populations in 176 cities. This study aimed to evaluate the role of social organizations in high-risk population interventions against HIV/AIDS.

**Methods:**

This study was based on the CAFNGO program from 2016 to 2020. The collected data included the number and types of social organizations participating in high-risk group interventions and the amount of funds obtained by these organizations each year. We explored the factors influencing the number of newly diagnosed AIDS cases using a spatial econometric model. Furthermore, we evaluated the effectiveness of intervention activities by comparing the percentages of the individuals who initially tested positive, and the individuals who took the confirmatory test, as well as those who retested positive and underwent the treatment.

**Results:**

Overall, from 2016 to 2020, the number of social organizations involved in interventions to protect HIV/AIDS high-risk populations increased from 441 to 532, and the invested fund increased from $3.98 to $10.58 million. The number of newly diagnosed cases decreased from 9128 to 8546 during the same period. Although the number of cities with overall spatial correlations decreased, the spatial agglomeration effect persisted in the large cities. City-wise, the number of social organizations (direct effect 19.13), the permanent resident population (direct effect 0.12), GDP per capita (direct effect 17.58; indirect effect − 15.38), and passenger turnover volume (direct effect 5.50; indirect effect − 8.64) were the major factors influencing new positive cases confirmed through the testing interventions performed by the social organizations. The initial positive test rates among high-risk populations were below 5.5%, the retesting rates among those who initially tested positive were above 60%, and the treatment rates among diagnosed cases were above 70%.

**Conclusions:**

The spatial effect of social organizations participating in interventions targeting high-risk populations funded by CAFNGO is statistically significant. Nevertheless, despite the achievements of these social organizations in tracking new cases and encouraging treatment, a series of measures should be taken to further optimize the use of CAFNGO. Working data should be updated from social organizations to CAFNGO more frequently by establishing a data monitoring system to help better track newly diagnosed AIDS cases. Multichannel financing should be expanded as well.

## Background

Acquired immunodeficiency syndrome (AIDS) is a serious global public health issue [1, 2]. According to the WHO, approximately 37.7 million people were living with human immunodeficiency virus (HIV) at the end of 2020, 680,000 died from HIV-related causes, and 1.5 million acquired HIV during the same year [1]. A study of AIDS epidemic trends in China from 1990 to 2017 found that after decades of prevention and control efforts, the AIDS incidence rate in China has decreased. However, AIDS-related mortality has risen significantly in recent times [2]; therefore, strengthening AIDS prevention remains an important task.

HIV/AIDS has become a social issue. Research on AIDS prevention and control has involved various areas of knowledge and a theoretical framework to help understand the epidemic from social, anthropological, biological, and psychological standpoints [3, 4]. In terms of AIDS, social organizations carry out interventions for high-risk populations to reduce the incidence of risky behaviors and care for infected individuals [5]. In addition to pushing for action to strengthen the social rights of AIDS-afflicted individuals, non-governmental organizations contribute to the rebuilding of the lives of these individuals [6]. Some social organization workers are members of high-risk groups or have contracted AIDS themselves and thus have natural advantages in such intervention and care work. They can establish better contact with and integrate into high-risk groups, thereby achieving notable outcomes in AIDS prevention and control [7]. A study in 2018 reported that social organizations play an important role in raising HIV/AIDS awareness and positively affecting the related behavior of migrants in China, helping advance HIV/AIDS prevention [8]. Silva et al. used descriptive statistical analysis to study the relationship between social support offered to HIV-infected individuals and social determinants of health. Social organizations’ participation in HIV/AIDS prevention and control not only plays a role in health education, prevention, screening, treatment, and defending the rights of HIV/AIDS patients but also promotes psychological support, family visits, social security, social assistance, medical education, and legal aid [9]. Moreover, social organizations are flexible and innovative. Recently, Internet-based detection services have significantly improved the accessibility and acceptance of HIV testing among the general population [10].

From 1980s, with China’s rapid urbanization, female sex workers (FSW), men who have sex with men (MSM), drug users (DU), young people, and the floating population have become high-risk AIDS populations in cities. Owing to the large number and high concealment of the population, implementing effective intervention by professionals alone is a challenge. However, social organizations that are intertwined and closely connected with these high-risk groups can effectively solve the above problems and compensate for the shortage of public health services from the government. Thus, social organizations have always played an important role in preventing and controlling AIDS. Since the early 1990s, with financial support from the Chinese government and international organizations, Chinese social organizations have begun to participate in preventing and controlling AIDS. International organizations have played a key role in supporting social organizations’ participation in AIDS prevention. According to a study conducted from 2005 to 2009, the proportion of international organization-supported funds for social organizations increased from 31.3 to 77.3% [11]. After 2010, international organizations successively stopped funding HIV/AIDS prevention and control measures in China. To continue with effective prevention and control, the Chinese government established the China AIDS Fund for Non-Governmental Organizations (CAFNGO) in 2015 to provide continued support to social organizations providing intervention and care services for high-risk groups. Now, 5 years after its implementation is useful to analyze its impact and contributions to AIDS prevention and control in China.

Spatial analysis involves the geographical or spatial location of an event as the input that corresponds to the spatial or geographical location of the analyzed subjects or events. Spatial epidemiology describes and analyzes geographically related indicators of health data, including demographic, environmental, behavioral, socioeconomic, genetic, and infection-related risk factors. Spatial analysis tools have significant advantages for investigating the incidence and risk factors of AIDS. Specifically, many studies on the spatial analysis of AIDS have focused on the spatial clustering and variation trends of AIDS. Kulldorff explored the practicality of spatial clustering [12]. Cuadros analyzed the spatial clustering and incidence trend of AIDS in Africa using this technique [13, 14]. Spatial clustering can provide a clearer understanding of the spatial specificity and regional differences in AIDS mortality [15, 16].

Moreover, the combination of hot/cold spot analysis and spatial clustering can better grasp the spatial transmission rules and dynamic changes in the time dimension. Global studies reported that the geographic coordinates of HIV-infected individuals could better predict the future cluster trends of HIV [17, 18]. Of the current epidemiological spatial analysis methods, maps are among the most powerful tools for describing spatial data. There are three basic types of disease maps: point-density maps, event maps, context diagrams of regional data, and isometric maps of continuous data. Other studies have reported that in the spatial analysis of HIV, different weighting methods to construct a spatial weight matrix may be more accurate for spatial clustering and statistical analysis, which can help with the precise prevention and control of AIDS, as well as resource allocation. Few studies have applied spatial econometric models to infer different types of risk factors in the spatial analysis of HIV. Some scientists used the Kriging interpolation method and Bayesian discriminant analysis for simulation and spatial optimization, but few on the risk factors using the spatial econometric model in the global scope. Thus, the current studies still have some limitations. It is noteworthy that many studies have focused on the dynamic changes in HIV/AIDS in different regions, but rarely on the positive role of social organizations in the intervention of HIV/AIDS prevention and control. Despite AIDS being a global disease, spatial research on AIDS interventions by social organizations in China remains limited.

This study explored the role of social organizations in HIV/AIDS interventions since their participation in the CAFNGO. Specifically, the study describes the measures taken by the fund, the number of social organizations involved, cost-effectiveness, and the quantitative relationships between the number of new AIDS patients and the number and funding of local social organizations.

## Methods

### Data collection

From January to March 2021, we desk-reviewed available CAFNGO program documents and related published articles to assess the measures taken and the progress and effect of the program over the past 5 years.

Next, we collected CAFNGO program data from 2016 to 2020 for each city, which included the number of social organizations involved in the program, the amount of funds obtained, the population of high-risk intervention recipients, the number of individuals who initially tested positive, the number of people who were retested after initially testing positive, the number of people who retested positive, and the number of those who tested positive and referred to treatment.

We focused on the social organizations supported by CAFNGO to provide interventions for high-risk populations: female sex workers (FSWs), men who have sex with men (MSMs), and drug users (DUs). The intervention service package included (1) mobilizing high-risk populations to be tested for HIV through peer education, outreach activities, etc.; (2) mobilizing those who tested positive to receive a confirmatory test; and (3) referring those who retested positive to treatment services. Most of these social organizations were established by members of high-risk populations, which was advantageous for providing intervention services.

Additionally, we collected confounding factors for 176 cities from the China Statistical Yearbooks (2017–2021), China Health Statistical Yearbooks (2017–2021), and Provincial Statistical Yearbooks (2017–2021), which included the number of practitioners in medical and health institutions, number of beds in medical and health institutions, permanent resident population, GDP per capita, educational expenditure, expenditure on health care and family planning, passenger turnover volume, population change in each city, per capita education spending, per capita medical spending, and incidence of AIDS in each province.

### Model establishment

A spatial econometric model was employed to analyze the relationship between the effectiveness of the CAFNGO program and engagement in social organizations.

The construction of exogenous spatial weights is a prerequisite for spatial autocorrelation analysis. Actual data from 2016 were selected. Using different spatial weight matrices and Monte Carlo simulations for robustness, Moran’s *I* was meaningful, indicating the existence of a spatial effect. The goodness-of-fit of the adjacency matrix was the best; therefore, the most widely used adjacency matrix was used (Table 1).

**Table 1:** The goodness of fit of different spatial weight matrices

Variable	Matrix type	Moran’s *I*	Monte-Carlo Simulation		
			*Z*	*SD*	*P*
Number of new HIV infections confirmed by intervention testing by social organizations in 2016	Adjacency matrix	0.127	3.9317	0.0332	0.009
	Geographically weighted matrix	0.134	3.6862	0.0371	0.013
	Euclidean distance matrix	0.023	1.9838	0.0140	0.040

*SD* Standard deviation

Moran’s indicator (Moran’s *I*) is widely used to verify the spatial dependence between items in spatial autocorrelation. In this study, Moran’s *I* was used to calculate the spatial autocorrelation of the number of new HIV-positive cases confirmed through social organization interventions. The global Moran’s *I* is defined as

1 $$I=\frac{n\sum_{i=1}^{n}\sum_{j=1}^{n}{\omega }_{ij}({\mathcal{X}}_{i}-\overline{\mathcal{X} })({\mathcal{X}}_{j}-\overline{\mathcal{X} })}{\sum_{i=1}^{n}\sum_{j=1}^{n}{\omega }_{ij}\cdot {\sum_{i-1}^{n}({\mathcal{X}}_{j}-\overline{\mathcal{X} })}^{2}}$$

where ${\mathcal{X}}_{\mathrm{i}}$ (${\mathcal{X}}_{\mathrm{j}}$) is the number of new AIDS cases in each prefecture-level city; i(j) is the city included in the study; ${\upomega }_{\mathrm{ij}}$ is the spatial weight matrix; and n is the number of cities included in the study. When *I* > 0, spatial autocorrelation presents agglomeration. When *I* < 0, spatial autocorrelation presents a discrete trend.

Spatial Gini coefficients are classified into three categories: dominant, recessive, and dominant-recessive Gini coefficients. Compared to the traditional relative Gini coefficient, they can more specifically analyze the aggregation degree and spatial structural dependence between items, defined as

2 $${\mathrm{G}}_{1}=\frac{1}{2{\mathrm{n}}^{2}{\upmu }_{{R}_{i}}}\sum_{\mathrm{i}=1}^{\mathrm{n}}\sum_{\mathrm{j}=1}^{\mathrm{n}}({R}_{i}-{R}_{j})$$

3 $${\mathrm{G}}_{2}=\frac{{\mathrm{R}}^{2}}{{\mathrm{n}}^{2}({\upmu }_{{R}_{i}}+{\upmu }_{\upomega {R}_{i}})}\sum_{\mathrm{i}=1}^{\mathrm{n}}\sum_{\mathrm{j}=1}^{\mathrm{n}}({R}_{i}-{\omega R}_{j})$$

4 $${\mathrm{Gini}=\mathrm{G}}_{1}+{\mathrm{G}}_{2}$$

where G1 represents the dominant spatial Gini coefficient, and G2 represents the recessive spatial Gini coefficient, which reflects the polarization effect of the spatial aggregation of the new number of AIDS cases. R is the determination coefficient of the spatial econometric model.

The general idea of spatial econometric analysis is to first test for spatial autocorrelation. Spatial autocorrelation analysis verified the spatial autocorrelation of the study subjects, which is a prerequisite for applying the spatial econometric model. There are three classic spatial models: spatial lag model (SLM), spatial error model (SEM), and spatial Durbin model (SDM). SLM, also known as the spatial autoregressive model (SAR), assumes that the explained variables are impacted by both locally explained variables and explained variables in the surrounding area. The term “lag” does not mean delay, but refers to a local variable interacting with the same variable in the surrounding area via feedback effects. SEM assumes that spatial dependence is the effect of neglected variables and is used to evaluate the influence of the error terms of the explained variables in the surrounding area on the local area. The SDM is a general form of the above two models, including endogenous and exogenous interactions, that is, the influence of the spatial lag terms of explanatory variables and the explained variables on the explained variables, is under consideration.

The study subject (explained variable) was the number of AIDS cases in each city, involving time and space. The following three spatial panel models were constructed based on the relevant variables:

1. Space panel lag model
  5 $${Y}_{it}=\rho W{Y}_{it}+{\alpha }_{0}+{\alpha }_{k}X{k}_{it}+{\varepsilon }_{it}$$
2. Spatial panel error model
  6 $${Y}_{it}={\alpha }_{0}+{\alpha }_{k}X{k}_{it}+{\xi }_{it}$$
  7 $${\xi }_{it}=\lambda W{\xi }_{it}+{\varepsilon }_{it}$$
3. Space panel Durbin model
  8 $${Y}_{it}=\rho W{Y}_{it}+{\alpha }_{0}+{\alpha }_{k}X{k}_{it}+{\beta }_{k}WX{k}_{it}+{\varepsilon }_{it}$$

where $\rho$ and $\lambda$ are the spatial correlation coefficients of the different models, $W$ is the spatial weight matrix, and ${\varepsilon }_{it}\sim N(0,{\sigma }^{2}I)$ is a random disturbance term.

Except for Moran’s *I*, spatial autocorrelation can be obtained using the Lagrange multiplier (LM) test. To verify the necessity of spatial econometric analysis and to select an appropriate model, the traditional non-spatial panel model (traditional econometric model) was analyzed using the LM test. The results showed that LM-Lag (12.40, *P* = 0.0297) and LM error (14.43, *P* = 0.0131) passed the significance tests. Thus, the factors influencing the change in the number of diagnosed patients include the spatial lag term and unobservable spatial autocorrelation error term of the explained variables, proving the necessity of the spatial panel model for analysis. According to the criterion proposed by Anselin and Florax [19], SDM is more appropriate. After determining whether to use the spatial panel model, it is also necessary to determine whether to use the random- or fixed-effect model. According to the results of the Hausman test, the hypothesis of there being no difference between the random- and fixed-effect model coefficients was rejected at the significance level of 5%. Thus, a fixed-effects model was used. The SDM model could not be simplified to the SLM (12.27, *P* = 0.0313) or SEM model (14.56, *P* = 0.0124); therefore, the SDM model was selected as the optimal model. An SDM with fixed effects was determined.

### Data analysis

Statistical analyses were performed using the Stata MP version 16.0. First, we describe the number, types, and spatial distribution of social organizations participating in high-risk group interventions and the amount of funds obtained by these organizations. Second, based on the description of the number and spatial distribution of newly diagnosed AIDS cases, a spatial econometric model was employed to analyze the relationship between the effectiveness of the CAFNGO program and the engagement of social organizations. The dependent variable was the number of newly diagnosed AIDS cases, which symbolized the effectiveness of the CAFNGO program, and the independent variables included the number of social organizations involved in the program and the amount of funds obtained. The number of practitioners in medical and health institutions, number of beds in medical and health institutions, permanent resident population, GDP per capita, educational expenditure, expenditure on health care and family planning, passenger turnover volume, population change in each city, per capita education spending, per capita medical spending, and AIDS incidence in each province were introduced into the model as covariates. We estimated the direct, indirect, and total effects of all independent variables and set a significance level of 0.05. Finally, we described three indicators in different groups and years, including the percentage of the population that initially tested positive, the percentage who tested positive and were retested for the confirmatory test, and the percentage who retested positive and received treatment, which reflected the ability and quality of the intervention service conducted by social organizations.

## Results

### Social organizations involved in high-risk groups intervention

#### The number of social organizations

From 2016 to 2020, the numbers of social organizations involved in AIDS intervention were 441, 539, 487, 516, and 632, respectively, of which 107, 160, 136, 196 and 208 were registered with the civil affairs department (see Table 2), accounting for 24.3%, 29.7%, 27.9%, and 38.0% and 39.1% of the total.

**Table 2:** Number of social organizations participating in intervention for high-risk groups from 2016 to 2020

	2016	2017	2018	2019	2020
Registered	107	160	136	196	208
Non-registered	334	379	351	320	324
Total	441	539	487	516	532

In 2017, the number of AIDS-related social organizations increased significantly as the project raised funds through social donations. However, the number of organizations decreased in 2018. Overall, the proportion of organizations that have completed registration in the civil affairs department shows an upward trend, suggesting that the project has played a significant role in cultivating the development of social organizations.

#### Spatial distribution of the number of social organizations

The degree of aggregation of social organizations participating in AIDS intervention testing is low, mainly in the eastern and central regions. A greater number of social organizations has been established in the more developed regions. In 2016, Chongqing and Beijing were extremely rich areas, with 21 and 23 social organizations, respectively, followed by Kunming, Qingdao, and Harbin, with 8, 13, and 7 organizations, respectively. The number of social organizations in Beijing and Chongqing increased to 25 and 21, respectively, in 2020. Overall, the total number of social organizations increased rapidly in the economically developed prefecture-level cities in the Southeast, which was positively correlated with the increase in the number of newly confirmed AIDS patients.

#### The number of social organizations participating in interventions for different high-risk population groups

The number of social organizations participating in interventions generally showed an upward trend. Notably, some organizations undertaking practical work may be involved in more than one type of group intervention; therefore, the sum of organizations involved in the three groups is not necessarily equal to the total number of organizations (Table 3).

**Table 3:** Number of social organizations participating in intervention for high-risk groups from 2016 to 2020

	2016	2017	2018	2019	2020
MSM	267	340	296	296	311
FSW	146	203	178	208	212
DU	66	76	62	74	74

*FSW* Female sex workers, *MSM* Men who have sex with men, *DU* Drug users

### Funds for conducting interventions in high-risk groups

#### The number of funds obtained by social organizations

From 2016 to 2020, special funds for AIDS prevention and control received by social organizations in Chinese yuan were 3.98 million, 4.93 million, 4.43 million, 10.06 million and 10.58 million USD, respectively, with an increase of 176.06% over the past 5 years and an average annual increase of 28.90%.

#### Spatial distribution of number of funds received by social organizations

In 2016, Beijing was regarded as an extremely rich area, meaning that the funds obtained by social organizations were significantly higher than those of other cities in China. Chongqing, Xi’an, Shanghai, Tianjin, and Harbin were also dense areas, and 37 other cities, such as Baotou, Panjin, and Huangshan, were extremely lacking in funds. In 2020, with the exception of Beijing, the funds obtained by social organizations in Shanghai sharply increased, and the city became an extremely dense area. Harbin, Chongqing and Qingdao were still dense areas, and 59 other cities, such as Lijiang, Wutong, Leshan, and Qujing, were extremely sparse areas. Generally, the funds of social organizations in China are gradually increasing, covering more areas. In the Central, Eastern, and Northwestern areas of China, funds increased more in economically developed areas than in less-developed areas. The growth trend in the amount of funds does not correspond to the growth trend in the number of social organizations. The increase in funds showed a clear tendency toward imbalance and was concentrated in the more economically developed central cities.

### The number of newly diagnosed AIDS cases and its influencing factors

#### Newly diagnosed AIDS cases

The numbers of newly diagnosed AIDS cases from 2016 to 2020 were 9129, 10,287, 8565, 9729 and 8546, respectively in each year, with a total of 46,256 individuals (Table 4). The MSM group accounted for the highest proportion, ranging from 97.0 to 98.4%.

**Table 4:** Newly diagnosed AIDS cases from 2016 to 2020

	2016	2017	2018	2019	2020
MSM	8903	9983	8361	9484	8406
FSW	90	206	142	159	97
DU	136	98	62	86	43
Total	9129	10,287	8565	9729	8546

*MSM* Men who have sex with men, *FSW* Female sex worker, *DU* Drug users

#### Spatial distribution of newly diagnosed AIDS cases

On the basis of the Jenks natural breaks classification, the areas with HIV-positive cases were classified into eight categories, where the blank areas were those with missing data, followed by extremely sparse areas, sparse areas, secondary sparse areas, equal areas, secondary dense areas, dense areas, and extremely dense areas (Table 5). In 2016, only Beijing and Chongqing were classified as extremely dense areas, but eight cities, including Harbin, Taizhou, and Chengdu, were classified as dense areas. In 2020, Beijing and Chongqing were extremely dense areas, and only Harbin and Chengdu were still dense areas, indicating that the number of dense areas had decreased. Newly diagnosed AIDS cases significantly increased in municipalities and provincial capitals, showing a tendency for cases to be concentrated in economically developed areas (see Annex.)

**Table 5:** Classification of newly diagnosed AIDS cases by intervention testing of social organizations in all 332 cities of China

Area	Number of cities and the proportion of population (%) in 2016	Number of cities and the proportion of population (%) in 2020
Blank area	156 (33.4)	156 (31.28)
Extremely sparse area	67 (17.6)	80 (20.1)
Sparse area	40 (12.8)	43 (15.4)
Secondary sparse region	31 (10.9)	22 (9.4)
Equal area	22 (9.6)	12 (6.0)
Secondary dense area	7 (5.4)	11 (7.2)
Dense areas	8 (8.8)	6 (6.7)
Extremely dense area	1 (1.6)	2 (3.9)

#### Spatial autocorrelation analysis of new HIV-positive cases

The local Moran scatter plot shows that most scatter points are in the first quadrant (high–high aggregation), that is, the spatial aggregation effect of the number of new AIDS cases in geographical space. From 2016 to 2020, Moran’s *I* gradually decreased, indicating that the agglomeration effect gradually decreased over the 5-year period. In 2020, Moran’s *I* declined, with a weakened spatial effect (Table 6).

**Table 6:** Monte Carlo simulation estimates of Moran *I* index

Year	Moran’s *I*	Z value	*SD*	*P* value
2016	0.111	3.6250	0.0233	0.010
2017	0.055	1.9483	0.0299	0.039
2018	0.024	0.9386	0.0329	0.088
2019	0.024	0.8403	0.0345	0.093
2020	0.030	0.9741	0.0318	0.085

*SD *Standard deviation

#### Positive spatial cluster analysis of newly diagnosed AIDS cases

The number of newly diagnosed AIDS cases was analyzed using *k*-means clustering. The results showed that Beijing was an extremely dense area, while Chongqing, Heilongjiang, and Zhejiang were dense in 2016. In 2017, Beijing remained an extremely dense area, and Jiangsu became a dense area. In 2018, Beijing was still an extremely dense area, Jiangsu was no longer a dense area, and Heilongjiang and Chongqing remained dense areas. In 2019, extremely dense areas remained unchanged, and Tianjin, Hebei, Jilin, Anhui, and Guangdong inchu dense areas. In 2020, Chongqing became an extremely dense area along with Beijing. The number of newly diagnosed AIDS cases in Anhui and Guangdong decreased; thus, they were no longer regarded as dense areas. The number of cases increased in Liaoning and Shaanxi, leading to dense areas. Overall, from 2016 to 2020, the number of newly diagnosed AIDS cases was concentrated mainly in economically developed areas such as Beijing, Tianjin, and Chongqing.

#### Spatial Gini coefficient

Spatial clustering can be used to describe similarities and differences between spatial units and adjacent spatial units. The spatial Gini coefficient (G) of the distribution of newly diagnosed AIDS cases from 2016 to 2020 was calculated. In 2016, the cities in Heilongjiang, Jilin, Hebei, Tianjin, Shaanxi, Sichuan, Hubei, Shanghai, and Guangdong were highly agglomerated, those in the northwest were blank, and those in the southern regions were low agglomerations, where the economic development level was weak and there was a lack of social institutions participating in AIDS prevention and treatment. Compared to 2016, the number of areas with high agglomeration of social institutions increased to 25 in 2020, with Tianjin, Hebei, and Chongqing as the centers. The Northwestern area remained blank, and the number of low agglomerations in the South decreased.

#### Influencing factors for the spatial distribution of newly diagnosed AIDS cases

The statistical results showed a significant correlation between the number of social organizations and the spatial distribution of new HIV-positive cases, as shown in Table 7. Specifically, every 1% increase in local social organizations increased the number of new HIV-positive cases by 19.13%. Every 1% increase in the permanent resident population of each city led to a 0.17% decrease in the number of new positive AIDS cases in the local area. Every 1% increase in GDP per capita led to an increase of 17.58% in new HIV-positive cases. However, every 1% increase in neighboring areas decreased the number by 15.35%. Every 1% increase in revenue passenger kilometers in each city led to an increase in the number of new HIV-positive cases by 5.50%. However, every 1% increase in neighboring areas decreased the number by 8.64% (Table 7).

**Table 7:** Spatial econometric results of newly diagnosed AIDS cases

Independent variable	Regression coefficient (β)	Spatial lag Wx	Direct effect	Indirect effect	Total effect
Number of social organizations	19.126***	− 4.843**	19.130***	− 0.685	18.445***
Funds received by social organizations (USD)	2.139	2.266	2.242	2.941	5.183*
Number of practitioners in medical and health institutions (people)	4.006	− 10.620	3.870	− 11.267	− 7.397
Number of beds in medical and health institutions	− 3.200	3.628	− 3.326	4.348	1.022
Permanent residents population of each city (10 thousand people)	0.116***	− 0.018	0.116***	0.006	0.122***
GDP per capita of each city (USD)	18.346***	− 17.097**	17.582***	− 15.377*	2.206
Educational expenditure of each city (10 thousand USD)	− 7.333	− 0.1176	− 7.421	− 1.539	− 8.961
Expenditure on health care and family planning of each city (10 thousand USD)	7.304	− 0.09294	7.393	1.560	8.963
Passenger Turnover Volume (10 thousand person-kilometers)	5.891***	− 8.842***	5.503***	− 8.636***	− 3.133
Population change of each city	− 0.045	− 0.576	− 0.048	− 0.076	− 0.125
Per capita education spending (USD)	7.359	− 0.135	7.445	1.524	8.970
Per capita medical spending (USD)	− 7.303	0.101	− 7.392	− 1.550	− 8.942
Incidence of AIDS in each province (1/100,000)	− 1.566	0.988	− 1.596	0.764	− 0.831

^***^P < 0.01, **P < 0.05, *P < 0.1

### Effect of interventions by social organizations

#### Percentages of individuals who initially tested positive

From 2016 to 2020, the preliminary positive screening rates were 5.5%, 5.3%, 4.7%, 4.4% and 4.1% respectively in MSM group;0.2%, 0.5%, 0.2%, 0.3% and 0.4% in FSW group; 0.5%, 0.6%, 0.4%, 0.4% and 0.4% in DU group. In total, the rate was higher for the MSM group, which meant that the AIDS epidemic in MSM was more severe than in other MSM and DU groups.

#### Percentage of the individuals who took the confirmatory test

The percentage of those who tested positive in the confirmatory test symbolized the ability of social organizations to keep track of those who were initially screened as positive. From 2016 to 2020, percentiges of individuals who took the confirmatory test were 88.2%, 88.1%, 89.6%, 88.5% and 79.8% respectively in MSM group; 76.1%, 50.8%, 70.4%, 66.0% and 29.1% in FSW group; 81.1%, 73.7%, 71.0%, 72.5% and 56.0% in DU group.

#### Percentages that retested positive and went through the treatment

The percentage that retested positive and went through the treatment was an indicator collected since 2017. All three groups showed an increasing trend from 2017 to 2020.  The persentages were 84.4%, 90.1%, 92.2% and 93.6% respectively in MSM group; 84.0%, 82.4%, 87.4% and 99.0% in FSW group;  72.5%, 82.3%, 97.7% and 95.4% in DU group (Table 8).

**Table 8:** Indicators for the effectiveness of intervention activities conducted by social organizations (%)

	2016	2017	2018	2019	2020	*χ*^2^	*P*
MSM							
Initially positive individuals positive	5.5	5.3	4.7	4.4	4.1	743.46	0.000
Individuals who took the confirmatory test	88.2	88.1	89.6	88.5	79.8	605.63	< 0.001
Those retested positive and went through the treatment	–	84.4	90.1	92.2	93.6	517.43	< 0.001
FSW							
Initially positive individuals positive	0.2	0.5	0.2	0.3	0.4	155.22	< 0.001
Individuals who took the confirmatory test	76.1	50.8	70.4	66.0	29.1	169.54	< 0.001
Those retested positive and went through the treatment	–	84.0	82.4	87.4	99.0	16.55	0.001
DU							
Initially positive individuals positive	0.5	0.6	0.4	0.4	0.4	16.98	0.002
Individuals who took the confirmatory test	81.1	73.7	71.0	72.5	56.0	23.49	< 0.001
Those retested positive and went through the treatment	–	72.5	82.3	97.7	95.4	27.58	< 0.001

*FSW* Female sex workers, *MSM* Men who have sex with men, *DU* Drug users

## Discussion

Through our spatial econometric model, we found that the spatial effect of social organizations participating in CAFNGO-funded interventions on high-risk groups was statistically significant. Although the number of cities with overall spatial correlations decreased, the spatial agglomeration effect persisted in the large cities. The number of social organizations, resident population, GDP per capita, and passenger turnover in each city were the main factors affecting the spatial distribution of new positive cases, as confirmed by the intervention testing performed by social organizations. Our results provide quantitative evidence for interventions and future strategy optimization for populations at a high risk of AIDS.

### Identification of AIDS cases facilitated by increasing the number of social organizations

Our case study was conducted in the context of social organizations independently supported by the Chinese government after the withdrawal of global funding. Spatial analysis showed that an increase in the number of social organizations in a city leads to an increase in the number of newly diagnosed AIDS cases in that city and a decrease in the number of newly diagnosed AIDS cases in adjacent areas. Therefore, social organizations play an important role in screening local high-risk groups. Because risk groups are also characterized by high mobility, an increase in the number of social organizations in one region can also promote testing among risk groups in neighboring regions. The important role of social organizations in HIV/AIDS prevention and control has already been confirmed in China and abroad [20–22]. Social organizations have rich experience working at the community level and act as a bridge between the community and the government. These organizations have promoted HIV testing through interventions while raising awareness of HIV/AIDS-related knowledge of high-risk groups [8, 23]. They also help the government track infected cases and patients, which generally helps to prevent the spread of HIV/AIDS. Therefore, social organizations play an important role in decreasing the incidence of HIV/AIDS.

Furthermore, the design of CAFNGO also promotes the engagement of social organizations. First, the number of social organizations that were registered in the civil affairs department increased steadily, which means that an increasing number of organizations operated regularly. In 2006, the Chinese government released the *Regulation on the Prevention and Treatment of HIV/AIDS,* which promoted the engagement of individual and social organizations in alleviating HIV/AIDS. It allowed social organizations to work without registration. However, more and more organizations were admitted and can run dependently and regularly. Second, CAFNGO allocated resources according to the prevalence of HIV, and consequently, funds were invested in those cities with a high prevalence of AIDS and organizations focusing on MSM interventions. Third, CAFNGO’s necessary performance measures help promote the efficiency of interventions by social organizations. For instance, CAFNGO purchases services according to the number of people participating in HIV testing instead of the number of interventions.

### Effective demographic and economic factors for social organization intervention

Urbanization, economic development, and the increase in the floating population are the factors that influence positive case identification. Our study found that an increase in the permanent resident population in each city led to a decrease in the number of new positive AIDS cases. An increase in GDP per capita leads to an increase in the number of new HIV-positive cases, while an increase in neighboring areas reduces the number. In addition, more people were diagnosed in economically developed cities, while fewer were diagnosed in the surrounding cities. One explanation may be that high-risk groups are more likely to move to densely populated and economically well developed areas. From 2016 to 2020, the number of newly confirmed AIDS cases screened by relevant social organizations displayed a decreasing trend overall, but an increasing trend in Beijing, Tianjin, and Chongqing, showing a tendency toward more economically developed areas. A series of studies have shown that AIDS-related social factors include economic status, education, employment, housing, physical and environmental exposure, and health spending [24, 25]. Although no correlation was found between the number of newly diagnosed AIDS cases and the variables in our study, such as expenditure on education, health care, and family planning, the incidence of AIDS in each province and economic and population factors may arouse public concern. A study in 2013 indicated that the incidence of AIDS is higher in cities with higher levels of economic wealth and urbanization. Rapid development in remote areas may be accompanied by an increased risk of AIDS [26]. This reflects that urbanization, economic development, and the increase in the floating population are factors influencing the high incidence of AIDS. The strength of social organizations in China is growing significantly, especially in developed cities, such as Beijing, Qingdao, Chongqing, and Chengdu. This growth was positively correlated with the increasing trend in the number of new HIV-positive cases.

### High-risk population prevention contributed by reasonable allocation of funds to social organizations

The funding received by social organizations in China is also increasing every year, with gradually expanding coverage. Economically developed cities, such as Beijing, Chengdu, Hangzhou, and Chongqing, have more funds than economically less-developed areas. However, funding growth was not positively correlated with an increase in the number of social institutions. Meanwhile, as the number of positive cases starts to decline, the efficient use of funds to purchase services from social organizations should be considered. Oliveira et al. analyzed an official report submitted by the Brazilian government to the Joint United Nations program on HIV/AIDS, which mentioned social welfare for adults infected with HIV/AIDS and performance-based incentives for HIV/AIDS prevention and control as one of the measures [24]. Although CAFNGO took measures to award organizations that could find more AIDS cases, most funds were paid according to the number of high-risk populations they could mobilize to test. Consequently, better performance regulation should be considered.

### CAFNGO’s assistance to social organization intervention

First, regional and high-risk group differences should be considered in detail for resource allocation. This study analyzed the data of Chinese social organizations participating in AIDS prevention and control projects during the recent 5-year period (from 2016 to 2020). The results showed that during this period, the number of newly diagnosed AIDS cases screened by social organizations presented a decreasing trend overall, but there was an increase in Beijing, Tianjin, and Chongqing, showing a tendency toward more economically developed areas. This indicates that urbanization, economic development, and the increase in the floating population are key factors influencing the high incidence of AIDS. The strength of social organizations in China is growing significantly, especially in developed cities such as Beijing, Qingdao, Chongqing, and Chengdu, which is positively correlated with the increasing trend in the number of new HIV cases; consequently, precise resource allocation is necessary.

Second, we proposed that the percentage of people who tested positive be retested and the percentage that tested positive a second time should undergo treatment, to evaluate the performance of social organizations in the future. These two indicators are innovative measures taken by CAFNGO. The decline in the percentage of those who tested positive in the confirmatory test was probably due to the COVID-19 pandemic, which restricted the work of social organizations. However, it is critical to track and manage infected cases or patients. Social organizations should make more efforts to track such individuals.

Third, informatization is an important measure to improve the effectiveness of CAFNGO. Currently, inaccurate or missing data remain a problem for related social organizations in China in determining the number of confirmed AIDS patients. Thus, it would be beneficial to build a big data platform for AIDS information collection and overall screening. A Brazilian study found that the Internet is a supplementary tool for people living with HIV/AIDS [27]. Some scholars believe that the widespread use of the Internet in developed countries gives patients and family members more access to diversified information sources. People often use the Internet for information inquiries using general search engines. In many cases, these searches are recorded before the visit and have been successfully extracted and used to predict seasonal influenza [28]. Thus, health sector decision-makers should establish a positive tracking strategy to track and contact confirmed AIDS patients to ensure that they receive effective treatment, strengthen the management of data, and implement intervention testing for real-time reporting. Such efforts are promoted by CAFNGO and may further promote the effectiveness of social organizations’ participation in AIDS intervention-related work. In terms of the current imbalance in the spatial distribution of social organizations, it is necessary to conduct a more detailed study to optimize spatial distribution and verify its effectiveness through simulation. In particular, resources should be re-allocated from economically developed to less-developed areas. The regional integration of fragmented high-quality resources can promote the development of infectious disease prevention and control in China. Simultaneously, human and financial inputs should be increased, with special attention paid to extremely dense areas. Because of the mobility of social work organizations, close attention should also be paid to the spatial effects of social organizations.

In this study, data from 2016 to 2020 were used. Thus, only a few years of data were selected, and the data continuity was weak, which may have resulted in deviations and bias. Since the construction of the spatial weight matrix was highly exogenous and the establishment time of social organizations in many regions was short, there may have been some limitations in constructing the spatial weight matrix. Finally, in terms of indicator selection, we selected large and numerous indicators, but there are not enough similar studies at present for comparison. The measurement indicators do not form a clear standard, which may result in some limitations. In the future, a more in-depth study will need to be carried out, especially to investigate the increasing intervention of social organizations at the county and district levels, striving to achieve full coverage and screening of AIDS cases, and reducing AIDS incidence. Moreover, we will explore more scientific research methods and perform more refined studies.

In addition, as data on new HIV infections discovered by the government through other channels cannot be obtained, as well as data before the launch of CAFNGO, the role of social organizations in the whole AIDS prevention and control system cannot be evaluated, which is also worth studying in the future.

## Conclusions

Despite the increasing challenges of HIV/AIDS, social organizations are important agents for limiting the impact of HIV/AIDS in China. A series of measures taken by CAFNGO, including expanding the engagement of social organizations, allocating resources rationally, and introducing a performance mechanism, also promote the engagement of social organizations and improve the efficiency of related interventions. We also believe that measures should be taken to promote social organizations’ engagement in HIV/AIDS prevention. First, building an efficient information system should be considered, which could help to reduce the number of duplicate tests and assess the performance of social organizations. Second, more attention should be paid to capacity building for social organizations, especially to improve their ability to track newly diagnosed AIDS cases and patients. Third, CAFNGO should attach importance to multichannel financing and continue to expand the regional participation of social organizations in AIDS prevention and control.

## Data Availability

The datasets generated and/or analyzed during the current study are inaccessible to peers because of confidentiality policies.

## Annex Number of newly diagnosed AIDS cases through social organizations intervention in 176 cities from 2016 to 2020

**Table:**

Year	Level	Cities	Value
2016	Extremely sparse area	Baotou, Panjin, Huludao, Hegang, Changzhou, Lianyungang, Zhoushan, Huangshan, Quanzhou, Yichun, Shangrao, Rizhao, Binzhou, Heze, Xuchang, Yichang, Xiangfan, Loudi, Yangjiang, Dongguan, Guilin, Wuzhou, Qinzhou, Guizhou, Yulin, Baise, Hechi, Chongzuo, Leshan Ya’an, Qujing, Zhaotong, Lijiang, Wenshanzhuangzu, Dalibaizuzizhizhou, Jinchang, Longnan, Xining, Zhongwei, Huzhou, Shiyan, Xiaogan, Zunyi, Zigong, Meishan, Honghe Hani And Yi, Suihua, Chuxiong, Weinan, Baiyin, Jiuquan, Yingkou, Qitaihe, Jiangmen, Benxi, Jixi, Guang An, Yuxi, Dehong Dai And Jingpo, Huaihua, Yi Chun, Hangzhou, Xuancheng, Zibo, Yongzhou	0–8
	Sparse area	Huainan, Xinyu, Dingxi, Chuzhou, Linyi, Xianning, Quzhou, Lishui, Weihai, Wuhan, Suizhou, Kaifeng, Hezhou, Nanchong, Anqing, Tai An, Jiaozuo, Panzhihua, Qinhuangdao, Shaoyang, Pingdingshan, Deyang, Wuwei, Tianshui, Zhangye, Zhenjiang, Liangshan Yi, Shangqiu, Datong, Suzhou, Jining, Suining, Wenzhou, Dezhou, Changde, Anshun, Tongchuan, Jingzhou, Yangzhou, Xuzhou	9–25
	Secondary sparse region	Nanchang, Guangyuan, Yibin, Zhoukou, Siping Weifang, Shuangyashan, Yancheng, Zhumadian, Hengyang, Qiqiher, Mudanjiang, Wuxi, Shaoxing, Liuzhou, Changzhi, Dalian, Ningbo, Dazhou, Wuhu, Lanzhou, Yinchuan, Sanmenxia, Maanshan, Zhuhai, Xinxiang, Guiyang, Daqing, Luoyang, Cangzhou, Taiyuan	26–53
	Equal area	Jiamusi, Hefei, Baoding, Nanning, Yantai, Zhanjiang, Taizhou, Jinan, Jinhua, Luzhou, Xingtai, Mianyang, Xi’An, Sanya, Langfang, Nanyang, Nanjing, Anshan, Fuzhou, Xiamen, Qingdao, Haikou	54–114
	Secondary dense area	Guangzhou, Kunming, Shijiazhuang, Zhengzhou, Suzhou, Zhangjiakou, Shenzhen	115–186
	Dense areas	Shanghai, Shenyang, Changsha, Changchun, Tianjin, Chendu, Harbin, Chongqing	187–434
	Extremely dense area	Beijing	435–1708
2017	Extremely sparse area	Suihua, Lianyungang, Yangzhou, Quzhou, Zhoushan, Binzhou, Xuchang, Xiaogan, Jiangmen, Yangjiang, Longnan, Loudi, Hezhou, Qujing, Yuxi, Lijiang, Jiuquan, Yichun, Jingzhou, Xianning, Nanchong, Ya’an, Zhaotong, Baiyin, Zhenjiang, Meishan, Dali Bai, Jinchang, Zhongwei, Huangshan, Xinyu, Suizhou, Yongzhou, Yulin, Hechi, Chongzuo, Suining, Dingxi, Jixi, Zigong, Wenshanzhuangzu, Weinan, Xuancheng, Rizhao, Shiyan, Anshun, Qitaihe, Huzhou, Lishui, Yichang, Shaoyang, Huaihua, Baise, Yichun, Dongguan, Honghe Hani And Yi, Huludao, Hegang, Weihai, Tianshui, Chuzhou, Taian, Baotou, Baoding, Jinan, Wuwei, Wuzhou, Qinzhou, Panzhihua, Guang An, Dehong Dai And Jingpo, Suzhou, Leshan, Zhangye, Qinhuangdao, Huainan, Linyi, Dezhou, Benxi, Weifang, Heze, Kaifeng, Yingkou, Wuhu, Guilin, Panjin, Xuzhou, Zibo, Guangyuan, Sanmenxia, Liangshan Yi, Anqing, Changde	0–20
	Sparse area	Quanzhou, Shangqiu, Xiangfan, Wuxi, Pingdingshan, Xining, Jiaozuo, Deyang, Mudanjiang, Zhanjiang, Chuxiong, Tongchuan, Siping, Changzhou, Hangzhou, Taizhou, Jinhua, Maanshan, Zhoukou, Wenzhou, Wuhan, Dazhou, Yancheng, Zhumadian, Hengyang, Shuangyashan, Shangrao, Changzhi, Lanzhou, Datong, Jining, Luoyang, Xinxiang, Shaoxing, Sanya, Guiguang, Luzhou, Zunyi, Daqing, Mianyang, Yinchuan	21–54
	Secondary sparse region	Yibin, Jiamusi, Dalian, Ningbo, Xingtai, Yantai, Kunming, Cangzhou, Nanyang, Qiqihar, Zhuhai, Xiamen, Anshan, Zhangjiakou, Taiyuan, Hefei, Langfang, Haikou, Zhengzhou	55–104
	Equal area	Guangzhou, Nanning, Nanchang, Suzhou, Fuzhou, Shijiazhuang, Qingdao, Guiyang, Xi’an	105–176
	Secondary dense area	Tianjin, Nanjing, Shenzhen, Shenyang, Chendu, Changchun,	177–358
	Dense areas	Changsha, Shanghai, Chongqing, Harbin	359–542
	Extremely dense area	Beijing	543–1318
2018	Extremely sparse area	Baoding, Suihua, Lianyungang, Yangzhou, Zhoushan, Quanzhou, Shangrao, Binzhou, Xuchang, Jiangmen, Yangjiang, Ya’an, Qujing, Lijiang, Baiyin, Dingxi, Longnan, Xiaogan, Xianning, Yuxi, Quzhou, Xinyu, Loudi, Zigong, Dali Bai, Jiuquan, Hezhou, Meishan, Zhaotong, Weinan, Yichun, Zhanjiang, Chongzuo, Guiyang, Jinchang, Tianshui, Baotou, Jixi, Yichun, Jingzhou, Zhongwei, Zhenjiang, Xuancheng, Jinan, Weifang, Wenshan Zhuangzuzhizhizhou, Hegang, Lishui, Huangshan, Chunzhou, Nanchong, Guang An, Rizhao, Yichang, Wuzhou, Yulin, Hechi, Panzhihua, Huzhou, Shiyan, Shaoyang, Dongguan, Liangshan Yi, Huaihua, Anshun, Benxi, Suizhou, Qinzhou, Honghe Hani And Yi, Yingkou, Huludao, Tai An, Weihai, Chuxiong, Dehong Dai And Jingpo, Guilin, Leshan	0–13
	Sparse area	Jiaozuo, Suining, Wuwei, Huainan, Linyi, Heze, Siping, Qitaihe, Changzhou, Shaoxing, Suzhou, Kaifeng, Pingdingshan, Qinhuangdao, Lanzhou, Xuzhou, Luoyang, Deyang, Zhangye, Xining, Changde, Panjin, Yancheng, Hengyang, Baise, Wenzhou, Zhumadian, Dazhou, Hangzhou, Zibo, Shangqiu, Guangyuan, Taizhou, Wuhu, Sanmenxia, Guigang, Tongchuan, Kunming, Yongzhou, Zunyi, Anqing, Wuhan, Dezhou	14–32
	Secondary sparse region	Maanshan, Zhoukou, Xiangfan, Daqing, Zhuhai, Yinchuan, Wuxi, Datong, Jining, Anshan, Changzhi, Shuangyashan, Qiqihar, Luzhou, Mianyang, Taiyuan, Xinxiang, Liuzhou, Dalian, Yantai, Mudanjiang, Jinhua	33–59
	Equal area	Zhangjiakou, Jiamusi, Guangzhou, Cangzhou, Zhengzhou, Xiamen, Yibin, Sanya, Nanyang, Nanning, Langfang, Ningbo	60–100
	Secondary dense area	Suzhou, Haikou, Nanjing, Hefei, Shijiazhuang, Fuzhou, Changchun, Nanchang, Tianjin, Shenzhen, Xi’an, Qingdao	101–212
	Dense areas	Shanghai, Shenyang, Chendu, Changsha, Harbin, Chongqing	213–525
	Extremely dense area	Beijing	526–1023
2019	Extremely sparse area	Lianyungang, Yangzhou, Zhenjiang, Shaoxing, Zhoushan, Binzhou, Xuchang, Shangqiu, Yangjiang, Panzhihua, Leshan, Ya’an, Qujing, Zhaotong, Jinchang, Jiuquan, Dingxi, Longnan, Xining, Yichun, Xuzhou, Quzhou, Xianning, Loudi, Meishan, Yuxi, Lijiang, Baiyin, Xiaogan, Yulin, Nanchong, Dali Bai, Weinan, Sanmenxia, Shiyan, Qinzhou, Hezhou, Zigong, Hegang, Hechi, Chongzuo, Suining, Yancheng, Xinyu, Zhangye, Baotou, Tai An, Linyi, Jixi, Lishui, Huangshan, Huaihua, Jiangmen, Dazhou, Honghe Hani, Wenshan Zhuangzuzizhizhou, Tianshuishi, Yichun, Wuzhou, Anshun, Dehong Dai And Jingpo, Zhongwei, Huzhou, Dezhou, Guang An, Chuzhou, Rizhao, Wuwei, Qitaihe, Yichang, Jingzhou, Guigang, Baise, Liangshan Yi, Suizhou, Yingkou, Ma Anshan, Suzhou, Weihai, Kaifeng, Pingdingshan, Chuxiong	0–14
	Sparse area	Suihua, Xuancheng, Weifang, Lanzhou, Benxi, Guangyuan, Huludao, Shaoyang, Kunming, Jinan, Luoyang, Huainan, Wenzhou, Zibo, Guilin, Anqing, Heze, Jiaozuo, Panjin, Qinhuangdao, Changzhou, Shangrao, Wuhan, Dongguan, Tongchuan, Hengyang, Xiangfan, Quanzhou, Wuxi, Wuhu, Zhumadian, Deyang, Yongzhou, Siping, Taiyuan, Zunyi	15–40
	Secondary sparse region	Yinchuan, Changde, Zhangjiakou, Taizhou, Zhuhai, Naning, Mianyang, Qiqihar, Hanghzou, Jinhua, Changzhi, Mudanjiang, Guangzhou, Yibin, Shuangyashan, Xinxiang, Zhoukou, Datong, Daqing, Yantai, Zhanjiang, Anshan, Liuzhou, Jiamusi, Xingtai, Sanya, Jining, Guiyang, Langfang, Cangzhou, Ningbo	41–85
	Equal area	Nanchang, Nanyang, Suzhou, Nanjing, Xiamen, Haikou, Dalian, Fuzhou, Zhengzhou, Baoding, Hefei	86–162
	Secondary dense area	Shijiazhuang, Tianjin, Changchun, Qingdao, Xi’an, Shanghai, Chengdu, Shenyang	163–333
	Dense areas	Shenzhen, Harbin, Changsha	334–525
	Extremely dense area	Beijing, Chognqing	526–718
2020	Extremely sparse area	Lianyungang, Yangzhou, Zhoushan, Binzhou, Xuchang, Shangqiu, Shiyan, Xiaogan, Loudi, Qinzhou, Ya’an, Qujing, Zhaotong, Lijiang, Jinchang, Baiyin, Jiuquan, Longnan, Xining, Yichun, Xuzhou, Dezhou, Xianning, Yangjiang, Hezhou, Zigogn, Nanchong, Meishan, Guang An, Yuxi, Dali Bai, Dehong Dai And Jingpo, Dingxi, Quzhou, Chongzuo, Suining, Weinan, Linyi, Sanmenxia, Yulin, Leshan, Wuwei, Baotou, Tai An, Panzhihua, Taiyuan, Jixi, Hechi, Anshun, Zhongwei, Huzhou, Lishui, Huangshan, Suzhou, Rizhao, Baise, Yancheng, Xinyu, Yichun, Jingzhou, Huaihua, Dazhou, Hegang, Qitaihe, Suihua, Kaifeng, Guigang, Honghe Hani, Wenshan Zhuangzuzizhizhou, Chuzhou, Suizhou Jingmen, Jinan, Guangyuan, Chuuxiong, Jiaozuo, Liangshan And Yi, Zhangye	0–12
	Sparse area	Zhenjiang, Luoyang, Pingdingshan, Xuancheng, Kunming, Lanzhou, Yingkou, Heze, Yichang, Tianshui, Huludao, Weifang, Shaoyang, Dong Guan, Wuzhu, Benxi, Wenzhou, Ma Anshan, Shangrao, Yongzhou, Anqing, Weihai, Hengyang, Deyang, Huainan, Zibo, Wuhan, Zhangjiakou, Wuxi, Zhanjiang, Panjin, Taizhou, Daqing, Datong, Mianyang, Tognchuan, Qinhuangdao, Guilin, Xiangfan, Changzhou, Quanzhou, Siping, Changde	13–34
	Secondary sparse region	Xinxiang, wuhu, Zhumadian, Guangzhou, Anshan, Shuangyashan, Nanning, inchuan, Qiqihar, Mudanjiang, Jinhua, Zhuhai, changzhi, Yantai, luzhou, Liuzhou, hanghzou, zhoukou, zunyi, xingtai, Guiyang, yibin	35–63
	Equal area	Sanya, Jiamusi, Shaoxing, Suzhou, cangzhou, Nanyang, Fuzhou, Xiamen, Ningbo, langfang, jining, baoding	64–120
	Secondary dense area	Nanjing, Haikou, Zhenghzou, Hefei, dalian, Nanchang, Changchun, Shenyang, Shijiazhuang, xi’An, Tianjin	121–233
	Dense areas	Qingdao, Harbin, Chengdu, Shanghai, Shenzhen, Changsha	234–418
	Extremely dense area	Beijing, Chongqing	419–628

## Abbreviations

AIDS: Acquired immunodeficiency syndrome
HIV: Human immunodeficiency virus
FSW: Female sex workers
MSM: Men who have sex with men
DU: Drug users
SLM: Spatial lag model
SEM: Spatial error model
SDM: Spatial Durbin model
SAM: Spatial autoregressive model
LM: Lagrange multiplier
